# Supplementary material for: The methyltransferase METTL9 mediates pervasive 1-methylhistidine modification in mammalian proteomes
Source: Nat Commun. 2021 Feb 9;12:891. doi: 10.1038/s41467-020-20670-7 (PMC7873184; doi:10.1038/s41467-020-20670-7)
Supplement: Supplementary file 1 — Supplementary Information [file 41467_2020_20670_MOESM1_ESM.pdf]

The methyltransferase METTL9 mediates pervasive 1-methylhistidine modification in mammalian proteomes

Erna Davydova*, Tadahiro Shimazu* et al. 2020

Supplementary Information

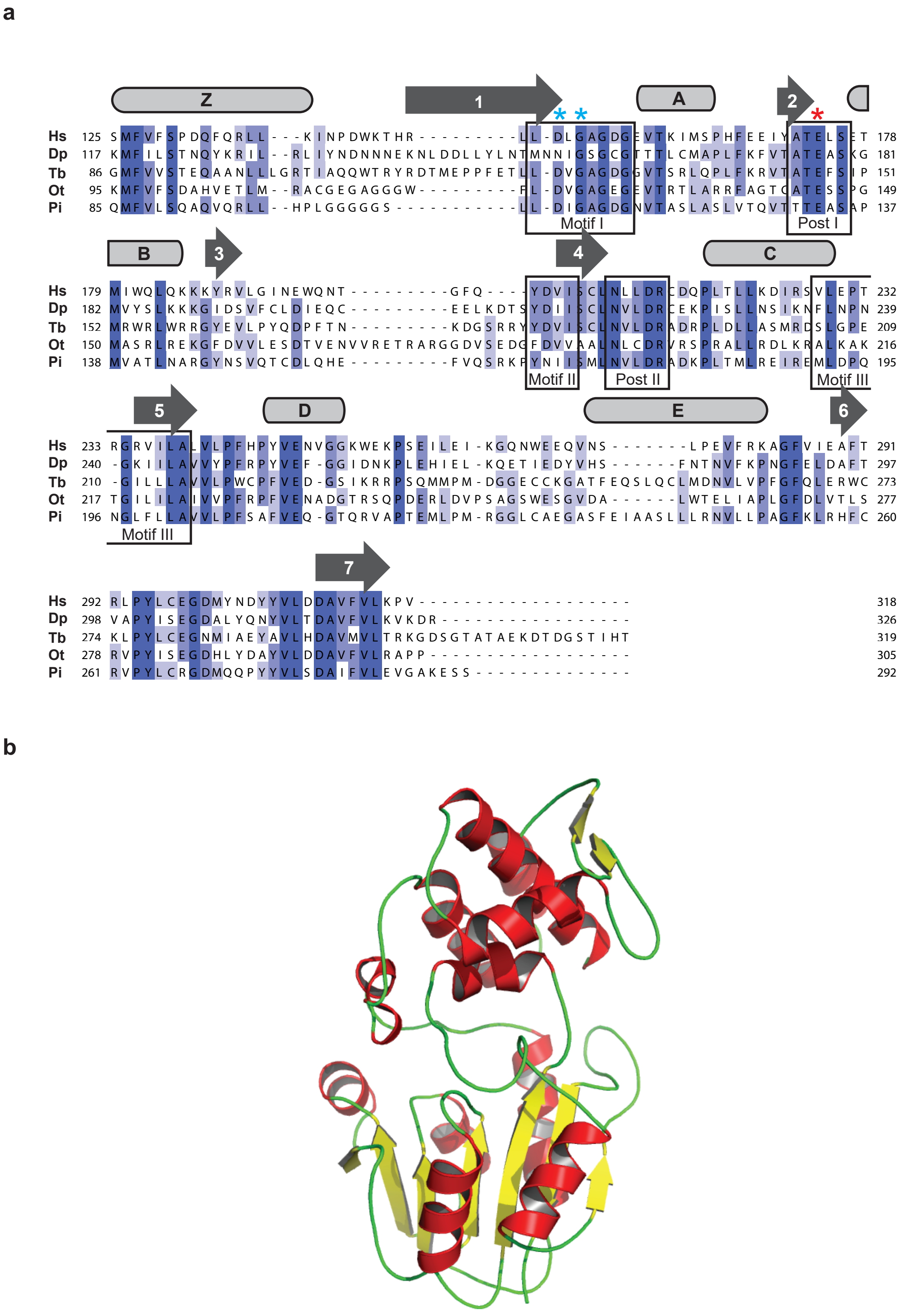

Supplementary Fig. 1: METTL9 is a eukaryotic 7BS methyltransferase.
a, Alignment of the core methyltransferase domain of METTL9 orthologues from *Homo sapiens* (Hs; Q9H1A3-1), *Dictyostelium purpureum* (Dp; XP_003287139.1), *Trypanosoma brucei* (Tb; AAZ11816), *Ostreococcus tauri* (Ot; Q01C52), and *Phytophthora infestans* (Pi; PITG_14565T0).
Predicted secondary structure is depicted as rectangles (α-helices) and arrows (β-strands). Hallmark 7BS-MTase motifs I-III and Post I-II are labelled and boxed. The glutamic acid residue mutated in the E174A hMETTL9 mutant is marked with a red asterisk, the aspartic acid and glycine mutated in the D151K/G153R mMETTL9 mutant are marked by blue asterisks. **b,** Predicted structure of human METTL9, α-helices in red and β-strands in yellow.

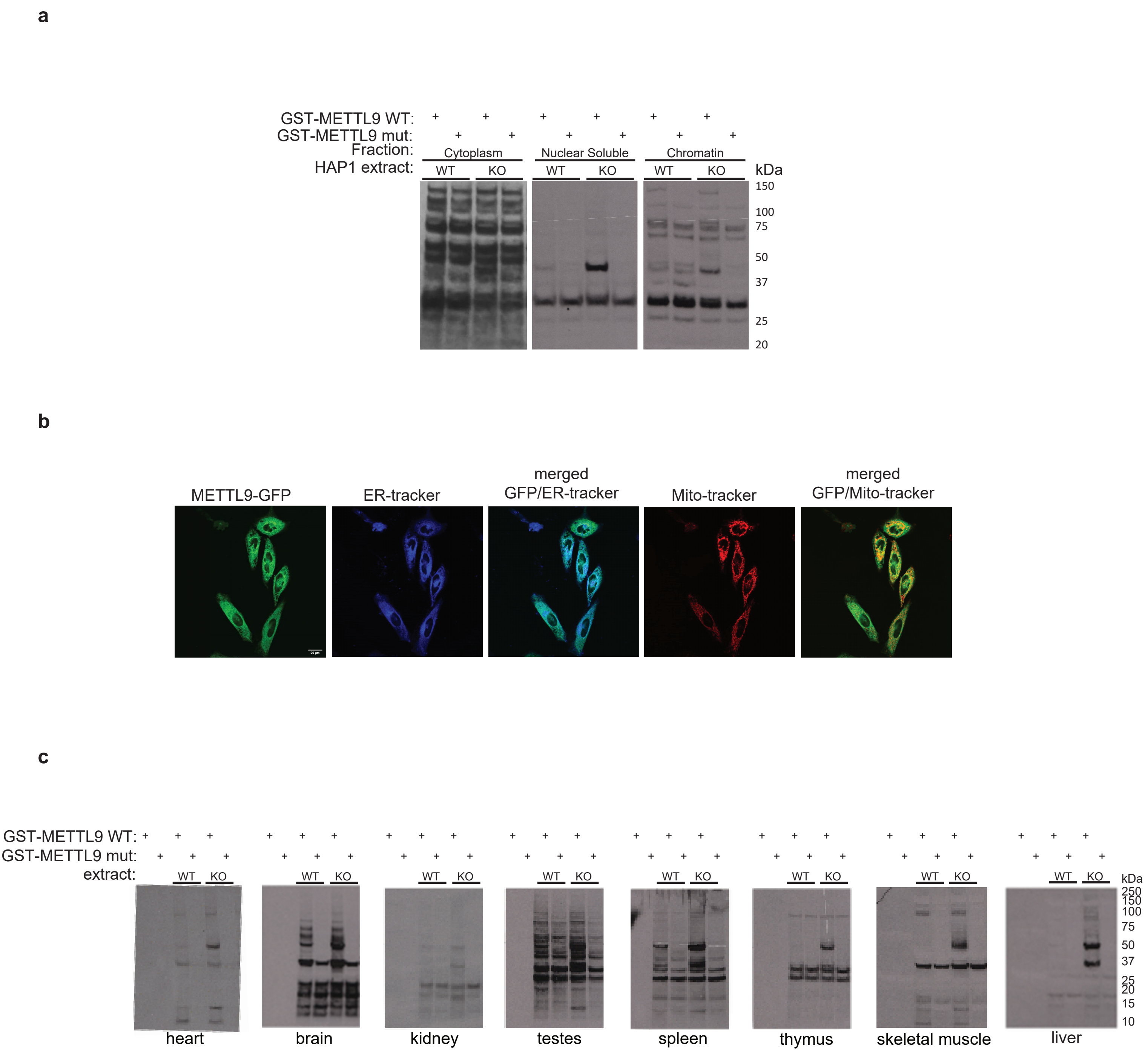

Supplementary Fig. 2: METTL9 is a protein methyltransferase present in different mammalian tissues and subcellular compartments. a, Fluorography showing the activity of wild-type GST-hMETTL9 (METTL9 WT) or the GST-hMETTL9 E174A inactive mutant (METTL9 mut) on cytoplasmic, nuclear soluble and chromatin-bound protein fractions from HAP1 WT or METTL9 knockout (KO) cells. **b,** Subcellular localization of hMETTL9-GFP in HeLa cells. Scale bar: 20 µm. **c,** Fluorography showing the activity of hMETTL9 WT or hMETTL9 mut on protein extracts from different tissues of WT or *Mettl9* KO mice. **a-c** show representative results of at least three independent experiments.

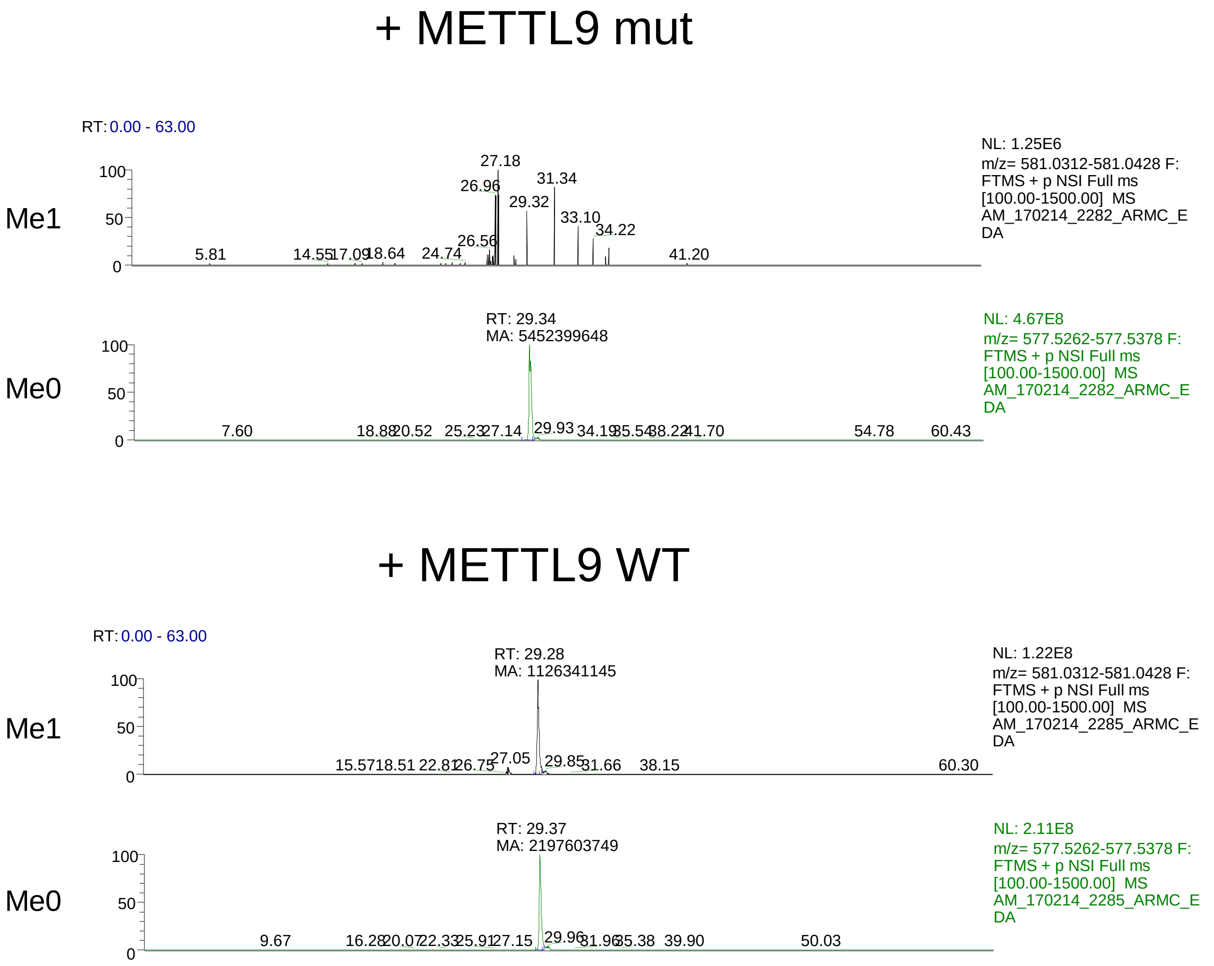

Supplementary Fig. 3: Extracted ion chromatograms (XICs) showing METTL9-mediated methylation of ARMC6 (detailed data for Fig. 1b). Recombinant His$_6$-ARMC6 was incubated with recombinant WT or mutant (E174A) His$_6$-hMETTL9 in the presence of AdoMet, and digested with trypsin. The resulting peptides were analysed by high resolution LC-MS. XICs corresponding to the methylated (Me1) and unmethylated (Me0) forms of the peptide VMTFDDDIRVPFGHAHNHAK (ARMC6$_{248\text{-}267}$) are shown. $z = 4$; theoretical m/z: 577.533 (Me0), 581.037 (Me1).

a

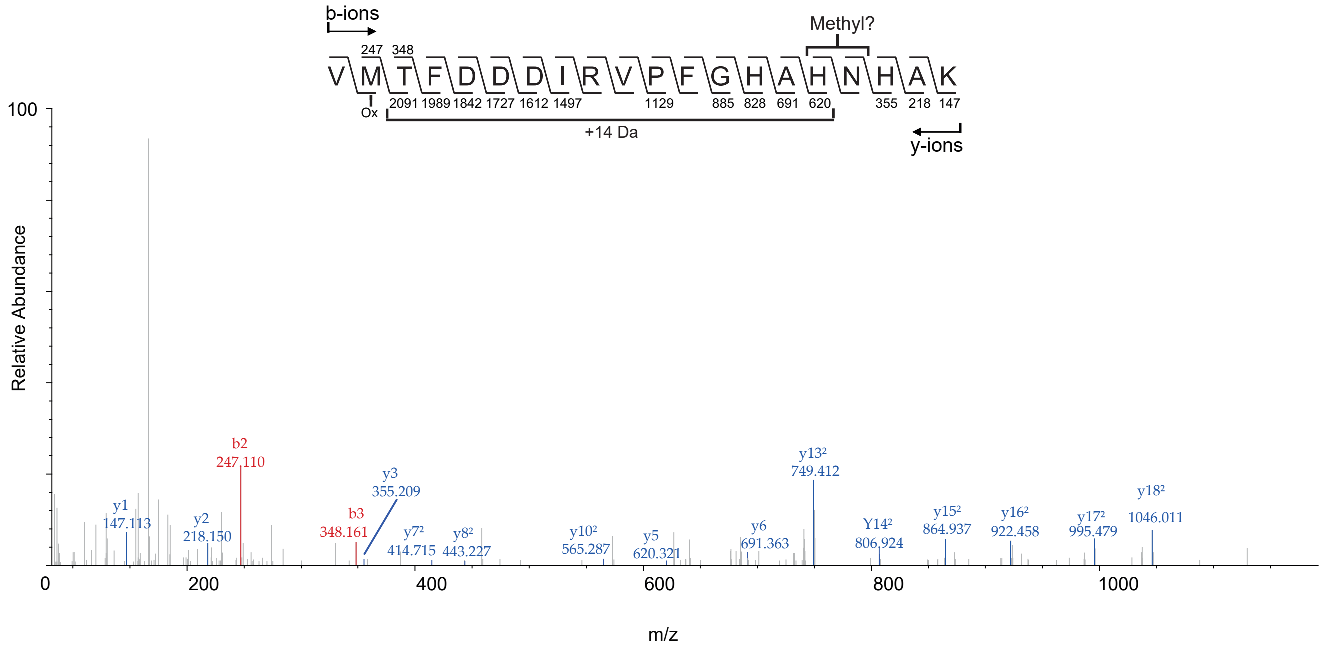

b

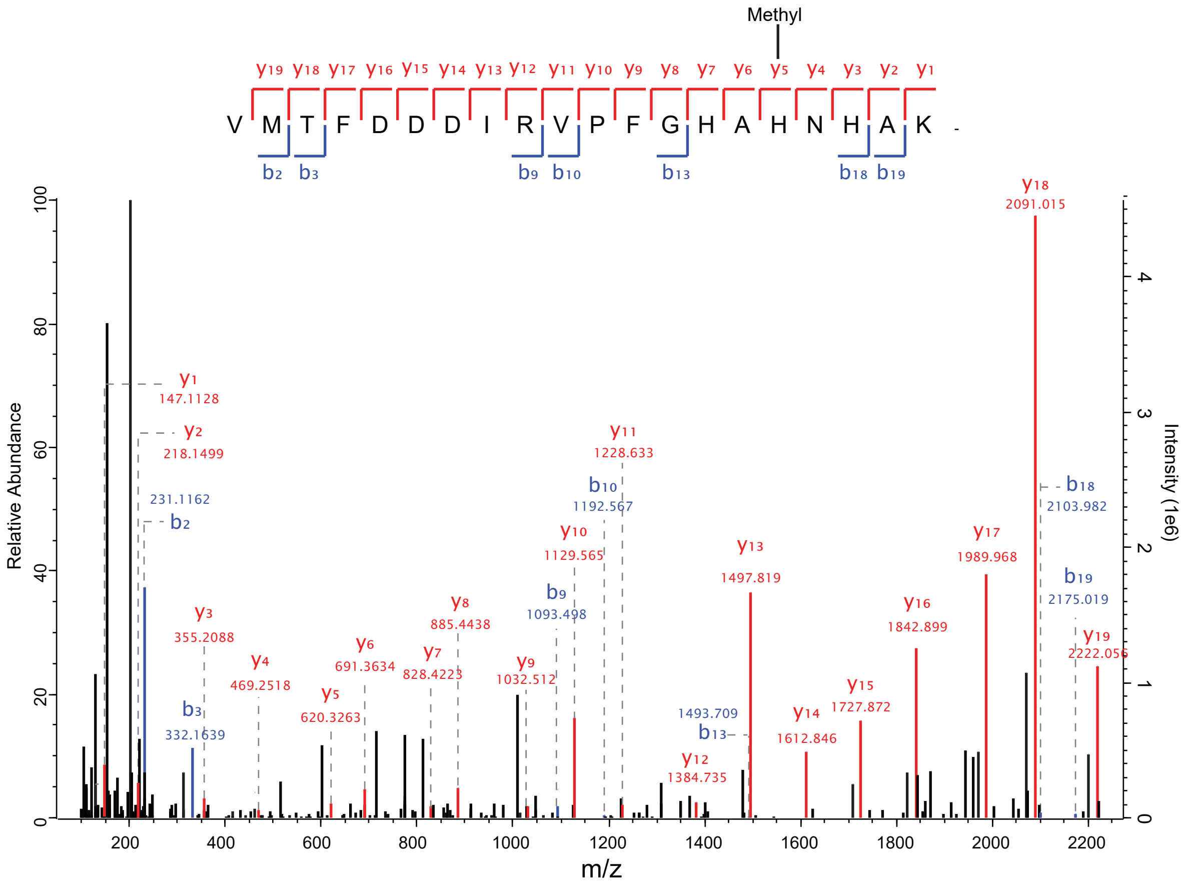

Supplementary Fig. 4: MS/MS evidence of ARMC6 His263 methylation.
a, MS/MS fragmentation spectra of a tryptic peptide from His_6-ARMC6 in vitro methylated with His_6-hMETTL9, indicating monomethylation at either His263 or Asn264 **b,** MS/MS fragmentation spectra of a peptide covering amino acids 248-267 in ARMC6 from Flp-In 293 T-REx cells pulled down with the hMETTL9-GFP bait, demonstrating monomethylation of His263.

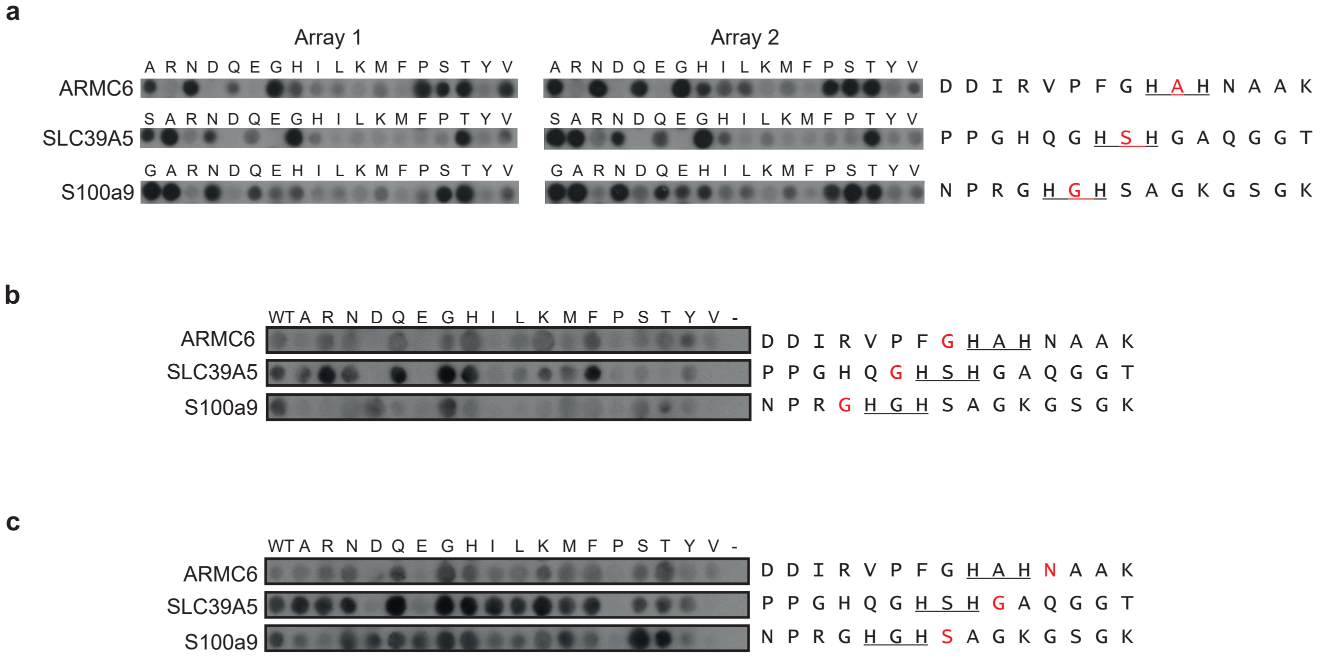

Supplementary Fig. 5: METTL9 activity on HxH peptide arrays.
a, Original peptide arrays used for quantification in Fig. 1f, showing the activity of His$_6$-hMETTL9 with [^{3}H]-AdoMet on peptides derived from ARMC6, SLC39A5 and S100a9 with replacements of the middle residue (red) in the HxH motif (underlined). Note that Array 1 is presented in Fig.1f rearranged to follow the same order of introduced mutations. Two independent peptide array experiments, each assessing three peptides, were performed. **b,** Similar to **a** but single array with replacements of the N-terminally flanking residue (red). **c,** similar to **b** but with replacements of the C-terminally flanking residue (red). The array experiments shown in **b** and **c** were only performed once, but with three different peptides. Thus, the main conclusions are supported by three independent experimental observations.

+ METTL9 mut

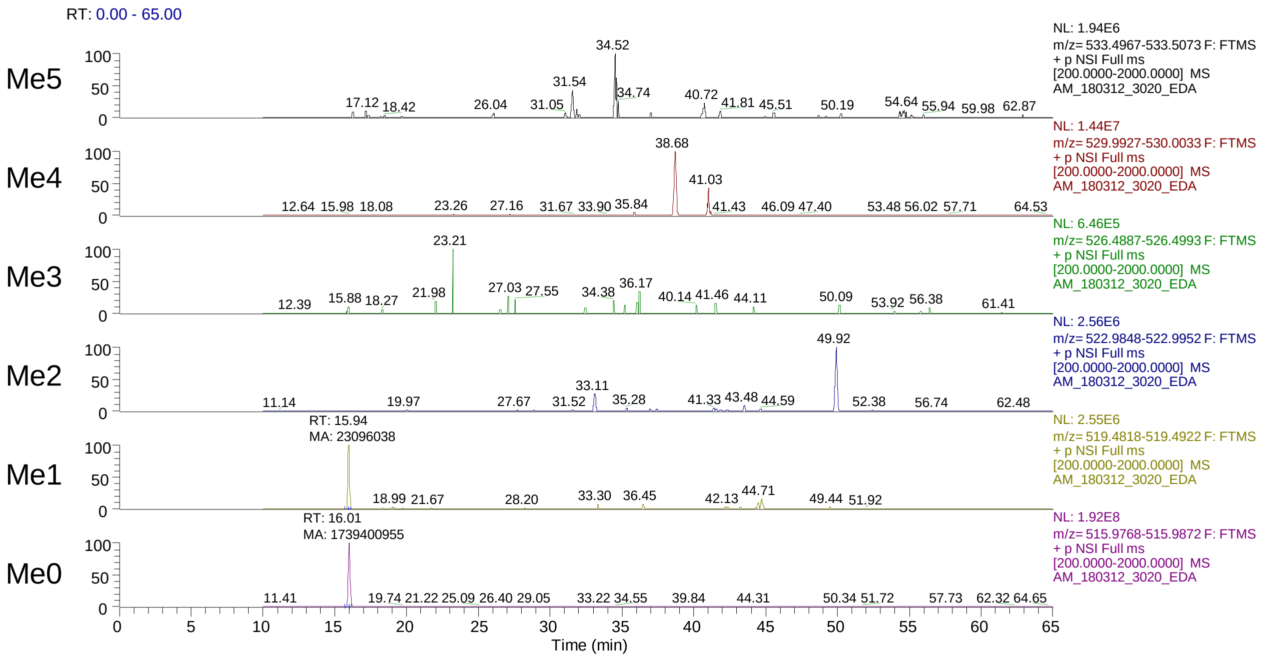

+ METTL9 WT

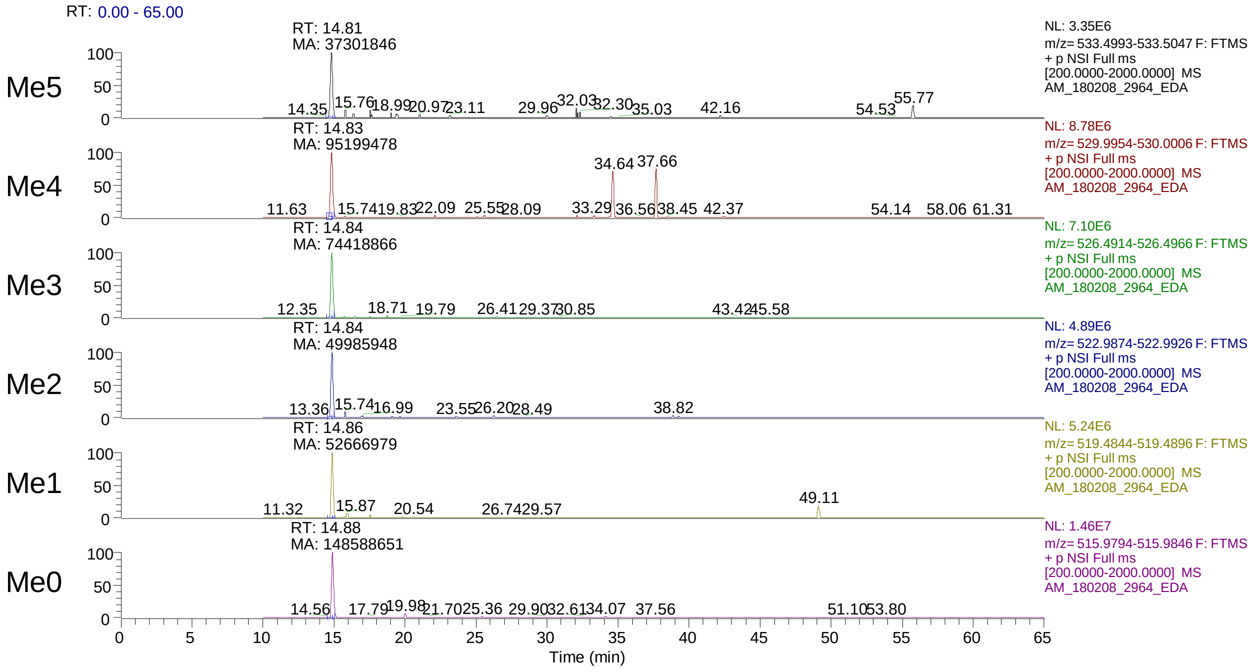

Supplementary Fig. 6: Extracted ion chromatograms (XICs) showing METTL9-mediated introduction of multiple methylations on a SLC39A7-derived fragment in vitro (detailed data for Fig. 1g). A recombinant fusion protein between GST and residues 31-137 of SLC39A7 (GST-SLC39A7$_{31\text{-}137}$) was incubated with recombinant WT or mutant His$_6$-hMETTL9 in the presence of AdoMet, and digested with chymotrypsin. The resulting peptides were analysed by high resolution LC-MS. XICs corresponding to different methylation states of the peptide HHGHSHAHGHGHTHESIW (SLC39A7$_{55\text{-}72}$) are shown. z=4; Theoretical m/z: 515.983 (Me0), 519.486 (Me1), 522.990 (Me2), 526.494 (Me3), 529.998 (Me4); 533.502 (Me5).

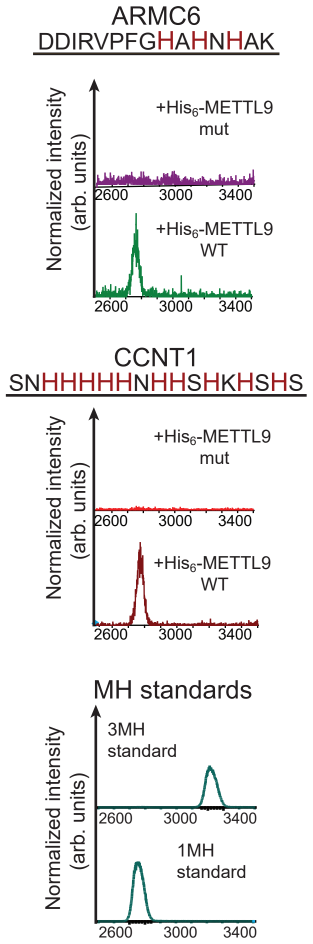

Supplementary Fig. 7: METTL9 forms 1MH in peptides derived from ARMC6 and CCNT1. Amino acid analysis of ARMC6 and CCNT1 peptides methylated by His_6-hMETTL9 WT or E174A inactive mutant (mut) as compared to the 3MH- and 1MH-methylhistidine standards. Shown are the normalized chromatograms with the elution times of the corresponding amino acid peaks.

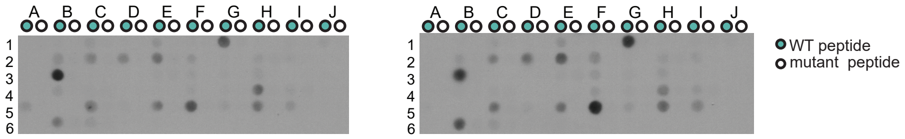

Supplementary Fig. 8: Peptide array both replicates. The peptide array experiments shown in Fig. 3e was performed twice with very similar results, which were used to generate Fig. 3d. Here, both arrays are shown (array on the right is presented in Fig. 3e).

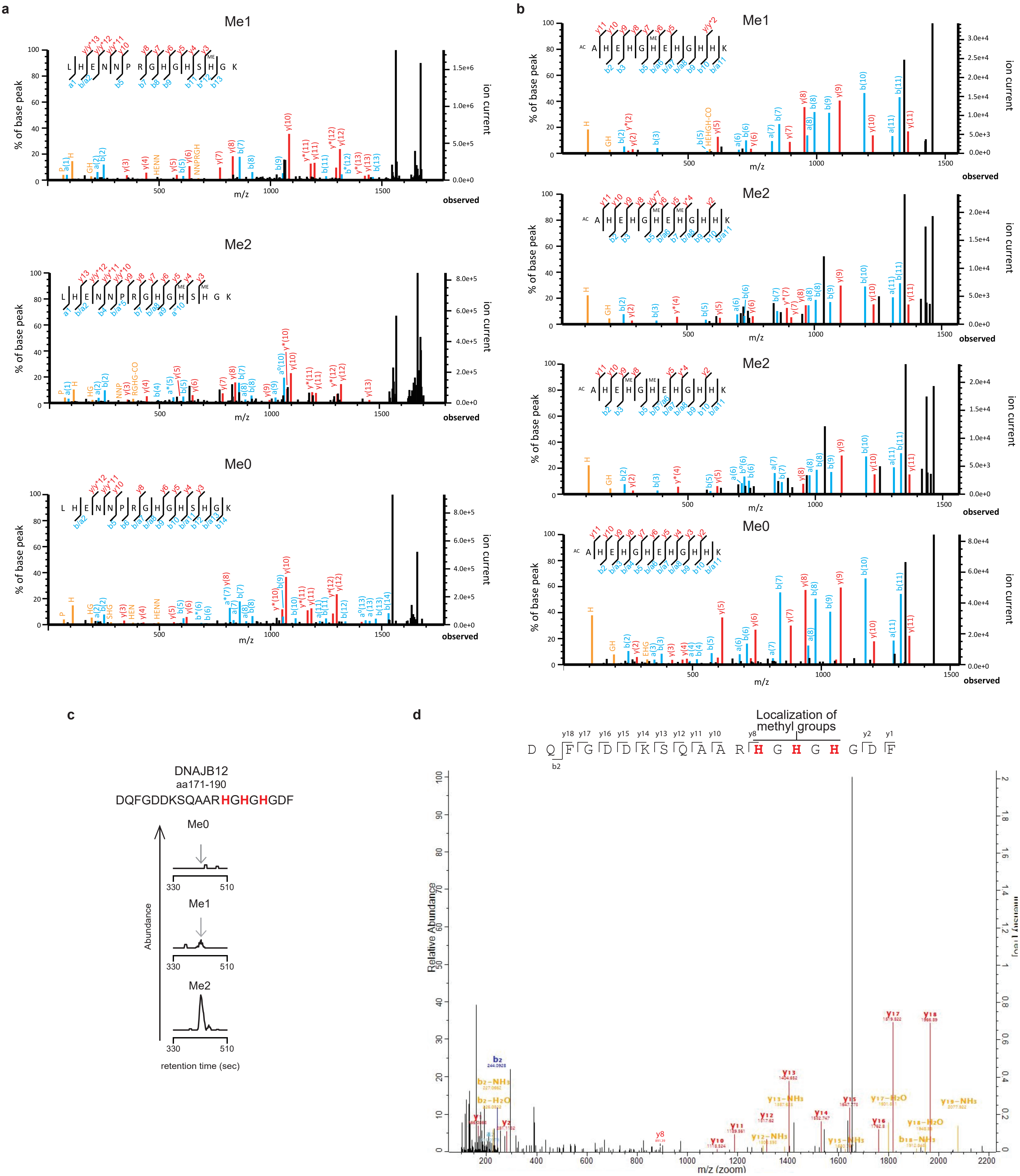

Supplementary Fig. 9: MS support of cellular METTL9-mediated histidine methylation.
a, MS/MS fragmentation spectra of a peptide covering amino acids 94-108 in S100A9 from peritoneal exudate neutrophils (PEN) isolated from WT mice indicating methylation at His106 (top); and methylation at His104/His106 (middle); or the unmethylated peptide from METTL9 KO mice (bottom). **b,** MS/MS fragmentation spectra of tryptic peptides from immunoprecipitated NDUFB3-FLAG from WT HEK293T cells indicating methylation at His6 (top); methylation at His6/His8 (second from top); methylation at His4/His6 (third from top); and no methylation (bottom). The middle panels show the same MS/MS spectra with different annotation corresponding to methylation at His6/His8 and His4/His6, respectively. **c,** Extracted ion chromatograms of chymotryptic peptides from endogenous DNAJB12 immunoprecipitated from HEK293T cells, with the indicated number of added methyl groups (Me0, Me1, Me2).**d,** MS/MS fragmentation spectra of the dimethylated DNAJB12 peptide suggesting methylation of histidines. Source data are provided as a Source Data file.

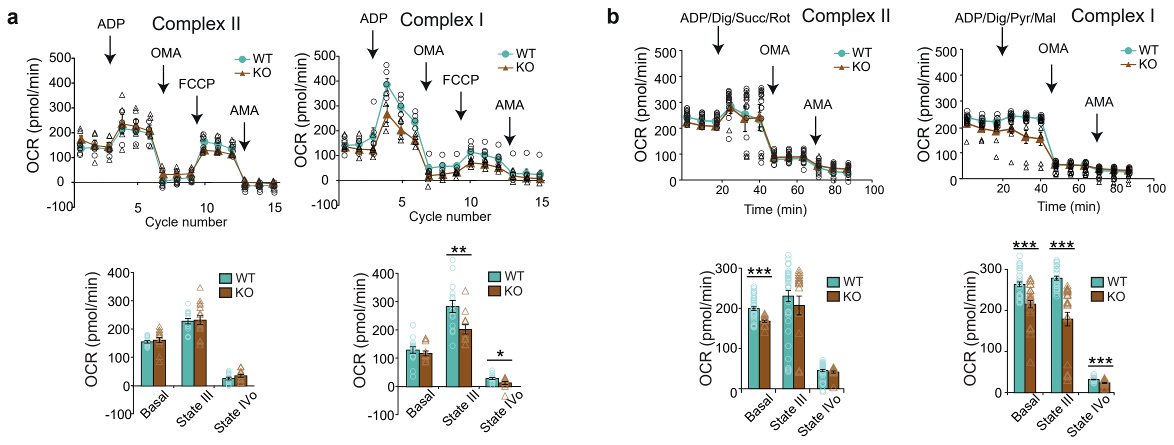

Supplementary Fig. 10: Mitochondrial respiration in WT vs METTL9 KO cells.
a, Oxygen consumption rate (OCR) of isolated mitochondria from WT or METTL9 knockout (KO) HAP1 cells (n = 5 (WT) or 4 (KO) biologically independent samples). Mitochondrial respiration driven by Complex II using succinate, in the presence of rotenone, as electron source (left panels) or Complex I using glutamate and malate (right panels). OCR traces (top) and quantification of respiratory states (bottom). Mean ± SEM. **P<0.01(P=0.0091);*P<0.05(P=0.0279); two-tailed Student's t-test. Source data are provided as a Source Data file. **b,** similar to a, but using digitonin-permeabilized WT or METTL9 KO HEK293T cells, with succinate/rotenone and pyruvate/malate as substrates for respiration mediated by Complex II (left; n = 13 (WT) or 6 (KO) biologically independent samples) and Complex I (right; n = 10 (WT) or 9 (KO) biologically independent samples), respectively. Mean ± SEM. ***P<0.001(Complex II: P=1.630e-04; Complex I: basal P=5.239e-05, state III P=2.564e-06, state IV P=2.710e-06); two-tailed Student's t-test. Source data are provided as a Source Data file.

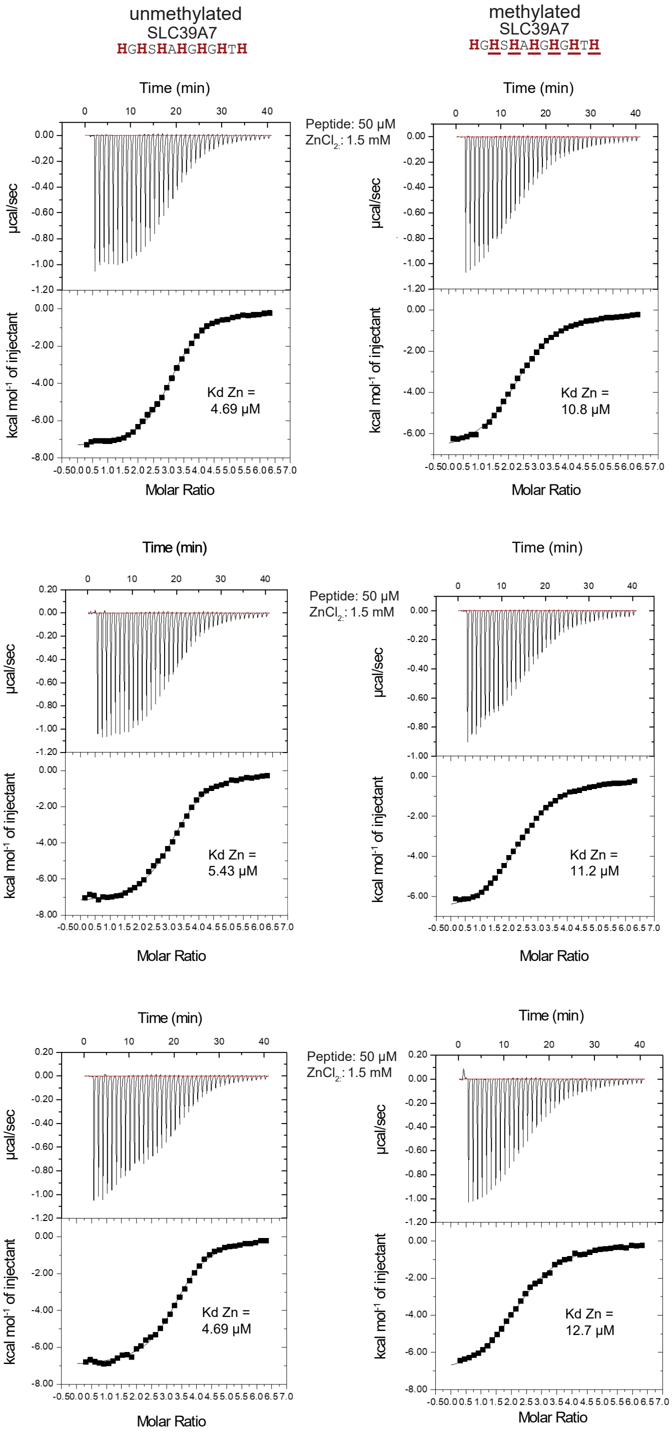

Supplementary Fig. 11: Determination of Zn^{2+} binding to methylated and unmethylated SLC39A7 peptides. Isothermal Titration Calorimetry measurements of the titration of $ZnCl_2$ binding to 50 µM unmethylated (left) or methylated (right, 1MH are underlined) SLC39A7 peptides. Heat changes (upper panels) and fitted data (lower panels) from three independent experiments.

Supplementary Table 1: Peptides used in the candidate substrate peptide array. Indicated are the position in the arrays from Fig. 3d (Pos), Uniprot ID and gene names, WT and control mutant peptide sequences and the corresponding average relative activity of METTL9, as well as the reasoning for inclusion of the peptides (Source) where "other" encompasses sequences reported as histidine-methylated in literature, putative methylhistidine-containing peptides identified through exploratory and further analysis of previously published HeLa proteomics datasets, various zinc transporters and other HxH-containing proteins of particular biological interest. Alternating histidines marked in blue, His-to-Ala mutations in red.

Pos	Uniprot ID	Gene	WT sequence	Mut sequence	WT avg relative activity	Mut avg relative activity	Source
A1	Q9H845	ACAD9	IRIGL RNHDH EVLLA	IRIGL RNADH EVLLA	0	0	Other
B1	Q92974	ARHGEF2	LISRI LQHSH GIEEE	LISRI LQASH GIEEE	0	0	Other
C1	Q6NXE6	ARMC6	RVPFG HAHNH AKMIV	RVPFG HAANH AKMIV	0.03	0	Interactants
D1		ARMC6	LTGAI THHGH HTDVV	LTGAI THAGH HTDVV	0	0	Interactants
E1	Q9NU22	MDN1	KLTQL ASGHS HGTFE	KLTQL ASGAS HGTFE	0.07	0	Interactants
F1	Q92614	MYO18A	LSSAI FKHQH KGGTL	LSSAI FKAQH KGGTL	0	0	Other
G1		MYO18A	SKPHH FLLGH SHGTN	SKPHH FLLGA SHGTN	0.68	0	Other
H1	O00159	MYO1C	VPTGE IIRVV HPHRP	VPTGE IIRVV APHRP	0	0	Other
I1		MYO1C	LEDTV KHHPH FLTHK	LEDTV KHAPH FLTHK	0	0	Other
J1	O94832	MYO1D	LNSKL GKHAH FSSRK	LNSKL GKAAH FSSRK	0.02	0	Other
A2	Q12965	MYO1E	QKLQM QIGSH EHFNS	QKLQM QIGSA EHFNS	0	0	Other
B2	Q7Z417	NUFIP2	YYFYN HSHNH HHHHH	YYFYN HSANH HAAHH	0.09	0	Other
C2		NUFIP2	PQHHH SHHHP HHHPQ	PQHHA SHHAP HHAPQ	0.19	0	Other
D2	Q8IY67	RAVER1	LSPGP NGHSH LLKVR	LSPGP NGASH LLKVR	0.26	0	Interactants
E2	Q13433	SLC39A6	LHHHH HQNHH PHSHS	LHHAA HQNHH PASHS	0.39	0	Interactants
F2	Q92504	SLC39A7	EDFHG HSHRH SHEDF	EDFHG ASHRA SHEDF	0.12	0	ProSeAM, interactants
G2	Q9ULF5	SLC39A10	DHSHS DGLHT IHEHD	DASHS DGLHT IAEHD	0	0	Interactants
H2	Q5T4S7	UBR4	HTSRS AYHSH KDQAL	HTSRS AYASH KDQAL	0.05	0.01	Other
I2	Q9NRG9	AAAS	PGPPP VLPHS PHSHL	PGPPP VLPHS PASHL	0.03	0	Other
J2	Q9NR19	ACSS2	AEAAV VGHPH PVKGE	AEAAV VGAPH PVKGE	0	0	Other
A3	Q8N187	CARF	TVNDI KNHIH EVQKS	TVNDI KNAIH EVQKS	0	0	Other
B3	Q9NXW2	DNAJB12	SQAAR HGHGH GDFHR	SQAAR HGAGH GDFHR	0.87	0	ProSeAM
C3		DNAJB12	SPRPS VGHIH RRVTD	SPRPS VGAIH RRVTD	0	0	ProSeAM
D3	Q6ZRV2	FAM83H	DFRFQ TSHFH RDQLY	DFRFQ TSAFH RDQLY	0	0	Other
E3	Q9H7Z3	NRDE2	EKKKK RKHQH HKKTK	EKKKK RKAQH HKKTK	0.01	0.01	Other
F3	O95263	PDE8B	TKNVH QSHSH LAMPI	TKNVH QSASH LAMPI	0.04	0	Other
G3	Q15149	PLEC	GGIID PVHSH RVPVD	GGIID PVASH RVPVD	0.01	0	Other
H3	Q15043	SLC39A14	QKNEH HHGHS HYASE	QKNEH HAGHS AYASE	0.04	0	Interactants
I3	Q9Y512	SAMM50	NKDVV VQHVH FDGLG	NKDVV VQAVH FDGLG	0	0	Other
J3	Q9UPW6	SATB2	AVSRL LAHQH PQAIN	AVSRL LAAQH PQAIN	0	0	Other
A4	Q9Y6X0	SETBP1	KHKEK QKHQH SEAGH	KHKEK QKAQH SEAGH	0	0	Other
B4	Q8TCJ2	STT3B	MALGN SRHGH HGPGA	MALGN SRAGH HGPGA	0.03	0	ProSeAM
C4	Q9C0I4	THSD7B	TFKHQ SYKAH HHSKS	TFKHQ SYKAA HHSKS	0.01	0	Other
D4	Q15643	TRIP11	TSALQ LEHEH LIKLN	TSALQ LEAEH LIKLN	0	0	Other
E4	P21397	MAOA	SDNII IETLN HEHYE	SDNII IETLN AEHYE	0	0	Other
F4	P51911	CNN1	LGEPA HNHHA HNYYN	LGEPA HNAAA HNYYN	0.04	0	ProSeAM
G4	O43676	NDUFB3	MAHEH GHEHG HHKME	MAHEA GHEAG HHKME	0	0	ProSeAM
H4	Q15427	SF3B4	GHPGH GHSHP HPFPP	GHPGH GASAP HPFPP	0.31	0	ProSeAM
I4		SF3B4	HGPHG LGHPH AGPPG	HGPHG LGAPH AGPPG	0.04	0	ProSeAM
J4	P08842	STS	LLVLS YLHVH TALFS	LLVLS YLAVH TALFS	0	0	ProSeAM
A5	Q8NEW0	SLC30A7	GHGHS HGSGH GHSHS	GHGAS HGSGH GASHS	0.05	0	ProSeAM, interactants
B5	P06702	S100A9	EGDEG PGHHH KPGLG	EGDEG PGAHH KPGLG	0	0	Other
C5	P31725	S100a9*	HENNP RGHGH SHGKG	HENNP RGHGA SHGKG	0.29	0	Other
D5	Q6ZMH5	SLC39A5	PGHQG HSHGH QGGTD	PGHQG HSAGH QGGTD	0.02	0	Interactants
E5	Q6P5W5	SLC39A4	GHSSH SHGGH SHGVS	GHSSA SHGGH SAGVS	0.27	0	Interactants
F5	Q9Y6M5	SLC30A1	SGHGH SHGGH GHGHG	SGHGA SHGGH GAGHG	0.81	0	Other
G5	Q8TAD4	SLC30A5	GHSDH GHGHS HGSAG	GHSDH GAGAS HGSAG	0.05	0	Other
H5	P48735	IDH2	KPITI GRHAH GDQYK	KPITI GRAAH GDQYK	0.33	0	Other
I5	A7E2V4	ZSWIM8	RLSPA HAHNH LRAPA	RLSPA HAANH LRAPA	0.13	0	Other
J5	Q14573	ITPR3	NQALL HKHLH LFLTP	NQALL HKALH LFLTP	0	0	Other
A6	Q6P087	RPUSD3	AQLPL HLHLH RLLLP	AQLPL HLALH RLLLP	0	0	Other
B6	O60563	CCNT1	NHHHH HNHHS HKHSH	NHHAA HNAHS AKHSA	0.38	0.01	Other
C6	O60583	CCNT2	HHTSS HKHSH SHSGS	HHTSS HKASH SASGS	0.06	0	Other
D6	P83436	COG7	MALLP HLHEH NLVKV	MALLP HLAEH NLVKV	0	0	Other
E6	P49773	HINT1	GQSVY HVHLH VLGGR	GQSVY HVALH VLGGR	0	0	Other
F6	Q9BX68	HINT2	AQSVY HLHIH VLGGR	AQSVY HLAIH VLGGR	0	0	Other
G6	Q9H1R3	MYLK2	AAARR GSPAF LHSPS	AAARR GSPAF LASPS	0	0	Other

*mouse protein

Supplementary Table 2: List of primers

Name	Sequence
pcDNA3-mMETTL9-F	AAAGAATTCACCATGAGACTGTTGGCGGGCTG
pcDNA3-mMETTL9-R	AAACTCGAGTACTGGTCTGAGAACAAAG
pET-mMETTL9-(22-)-F	AAACATATGTGGACGCTGCGGAGCCCGCTC
pET-mMETTL9-R	AAACTCGAGTTATACTGGTCTGAGAACAAAG
pQC-mMETTL9-cHA-F	AAAACCGGTACCATGAGACTGTTGGCGGGC
pQC-mMETTL9-cHA-R	AAAGGATCCCTAGATAGCGTAATCTGGAAC
mMETTL9-seq541	TGGCAGCTCCAGAAGAAGAA
mMETTL9-D151K/G153R-F	TTCTTAAGTTACGTGCTGGAGATGGAGAAGTC
mMETTL9-D151K/G153R-R	CCAGCACGTAACTTAAGAAGTCTATGGGTTTTC
pFBHT-Linearized vector-F	TAATGAGCCATGGGATCCGGAATT
pFBHT-Linearized vector-R	GCCCTGAAAATACAGGTTT
pFBHT-mMETTL9-22-F	CTGTATTTTCAGGGCTGGACGCTGCGGAGCCCGCTCT
pFBHT-mMETTL9-318-R	TCCCATGGCTCATTATACTGGTCTGAGAACAAA
pEGFP-N1_hMETTL9_F	GACTCAGATCTCGAGATGAGACTGCTGGCGGGC
pEGFP-N1_hMETTL9_R	GCGACCGGTGGATCCCGTACTGGTTTGAGAACAAAGACAGCGTC
pET28a_hMETTL9_F	GTGCCGCGCGGCAGCCATATGAGACTGCTGGCG
pET28a_hMETTL9_R	GTGGTGGTGGTGGTGCTCGAGTTATACTGGTTTGAGAACAAAGAC
hMETTL9_E174A_F	GAAATCTATGCCACTGCGCTTTCTGAAACTATG
hMETTL9_E174A_R	CATAGTTTCAGAAAGCGCAGTGGCATAGATTTC
pGEX-6P-2_hMETTL9_F	AGTCACGATGCGGCCGCTTATACTGGTTTGAGAACAAAGA
pGEX-6P-2_hMETTL9_R	AATTCCCGGGTCGACTATGAGACTGCTGGCGGGC
p3xFLAG-CMV-14_hMETTL9_F	AAGCTTGCGGCCGCGGCCACCATGAGACTGCTGGCG
p3xFLAG-CMV-14_hMETTL9_R	GTCAGCCCGGGATCCTACTGGTTTGAGAACAAAG
pET28a_ARMC6_F	GTGCCGCGCGGCAGCCATATGAGTGAACGATGTTGCTCTAGATACAG
pET28a_ARMC6_R	GTGGTGGTGGTGGTGCTCGAGTCATGGCGCCAGGTTGC
pET28a_ARMC6_H261R_F	CATGGTTGTGGGCACGGCCAAAGGGCACA
pET28a_ARMC6_H261R_R	TGTGCCCTTTGGCCGTGCCCACAACCATG
pET28a_ARMC6_A262G_F	GGCATGGTTGTGGCCATGGCCAAAGGG
pET28a_ARMC6_A262G_R	CCCTTTGGCCATGGCCACAACCATGCC
pET28a_ARMC6_H263R_F	ATCTTGGCATGGTTGCGGGCATGGCCAAAGG
pET28a_ARMC6_H263R_R	CCTTTGGCCATGCCCGCAACCATGCCAAGAT
pET28a_ARMC6_N264D_F	ATCATCTTGGCATGGTCGTGGGCATGGCCAAAG
pET28a_ARMC6_N264D_R	CTTTGGCCATGCCCACGACCATGCCAAGATGAT
pET28a_ARMC6_H265A_F	TTGGCCATGCCCACAACGCTGCCAAGATGATTGTGC
pET28a_ARMC6_H265A_R	GCACAATCATCTTGGCAGCGTTGTGGGCATGGCCAA
pET28a_ARMC6_A266G_F	CTGCACAATCATCTTGCCATGGTTGTGGGCATG
pET28a_ARMC6_A266G_R	CATGCCCACAACCATGGCAAGATGATTGTGCAG
pET28a_ARMC6_K267R_F	TTCTCCTGCACAATCATCCGGGCATGGTTGTGGGCATG
pET28a_ARMC6_K267R_R	CATGCCCACAACCATGCCCGGATGATTGTGCAGGAGAA
pGEX-6P-2_SLC39A7_31-137_F	GGGCCCCTGGGATCCCATGACGACCTGCACG
pGEX-6P-2_SLC39A7_31-137_R	ATGCGGCCGCTCGAGCTAATAAGCCCAGAGAGTGACAGC
pGEX-6P-2_DNAJB12_1-243_F	GGGCCCCTGGGATCCATGTCATCACTCCGCGC
pGEX-6P-2_DNAJB12_1-243_R	ATGCGGCCGCTCGAGCTACCCGCCATCACCCTG
pGEX-6P-2_DNAJB12_1-243_H185R_F	CCGGCACGGCCGTGGGCATGGGG
pGEX-6P-2_DNAJB12_1-243_H185R_R	CCCCATGCCCACGGCCGTGCCGG
pGEX-6P-2_CCNT1_F	GGGCCCCTGGGATCCATGGAGGGAGAGAGGAAGAAC
pGEX-6P-2_CCNT1_R	ATGCGGCCGCTCGAGTTACTTAGGAAGGGGTGGAAGTG
pGEX-6P-2_CCNT1_H519R/H520R/H523R/H524R/H528R_F	CCATCTAATCATCATCGTCGTCATAATCGCCGCTCACACAAGCGCTCTCATTCCCAACTTC

pGEX-6P-2_CCNT1_H519R/H520R/H523R/H524R/H528R_R	GAAGTTGGGAATGAGAGCGCTTGTGTGAGCGGCGATTATGACGACGATGATGATTAGATGG
pGEX-6P-2_hNDUFB3_F	GGGCCCCTGGGATCCATGGCCCATGAACATG
pGEX-6P-2_hNDUFB3_R	ATGCGGCCGCTCGAGTCAGTGATGCTTCTTATCTTTATTC
pGEX-6P-2_hNDUFB3_H5R/H9R_F	GGGCCCCTGGGATCCATGGCCCATGAACGTGGACATGAGCGTGGACATCATAAAATG
pcDNA3-hNDUFB3-F	AAAGAATTCACCATGGCCCATGAACATGGAC
pcDNA3-hNDUFB3-R	AAAGCGGCCGCCAGTGATGCTTCTTATCTTTA
pQC-hNDUFB3-cFLAG-F	AAAACCGGTACCATGGCCCATGAACATGGAC
pQC-hNDUFB3-cFLAG-R	AAATTAATTAACTAGAGCTTGTCATCGTCGTC
gRNA-hMETTL9-Intron2#1-F	CACCGTAATAAGTGATTATGGTTGT
gRNA-hMETTL9-Intron2#1-R	AAACACAACCATAATCACTTATTAC
gRNA-hMETTL9-Intron3#2-F	CACCGCAGTATTTTTCTGGAGCGG
gRNA-hMETTL9-Intron3#2-R	AAACCCGCTCCAGAAAAATACTGC
hMETTL9-genotype-F	GATCACGAGGGCAGGAGAT
hMETTL9-genotype-R	GTTGTCAGGGGTTACGAGGA
