# Supplementary material for: The methyltransferase METTL9 mediates pervasive 1-methylhistidine modification in mammalian proteomes
Source: Nat Commun. 2021 Feb 9;12:891. doi: 10.1038/s41467-020-20670-7 (PMC7873184; doi:10.1038/s41467-020-20670-7)
Supplement: Supplementary file 3 — Description of Additional Supplementary files [file 41467_2020_20670_MOESM3_ESM.docx]

**Description of Additional Supplementary Files**

File Name: Supplementary Data 1

Description: Table showing experimental and calculated masses, as well as ppm deviation, for MALDI MS experiments (Fig. 4 b,c)

File Name: Supplementary Data 2

Description: MASCOT search results for MALDI MS experiments (Fig. 4 b,c)
