# Supplementary material for: The methyltransferase METTL9 mediates pervasive 1-methylhistidine modification in mammalian proteomes
Source: Nat Commun. 2021 Feb 9;12:891. doi: 10.1038/s41467-020-20670-7 (PMC7873184; doi:10.1038/s41467-020-20670-7)
Supplement: Supplementary file 5 — Supplementary Data 2 [file 41467_2020_20670_MOESM5_ESM.pdf]

## Supplementary Data 2

MASCOT search results for MALDI MS experiments showing in vivo methylation of mouse S100A9 and human NDUFB3 (supporting data for Fig. 4b,c)

# hNDUFB3 - WT cells

# MATRIX SCIENCE MASCOT Search Results

## Protein View: hNDUFB3

### hNDUFB3

| | |
|---|---|
| **Database:** | Shimazu |
| **Score:** | 48 |
| **Expect:** | 3.2e-005 |
| **Monoisotopic mass ($M_r$):** | 13052 |
| **Calculated pI:** | 7.30 |

Sequence similarity is available as an NCBI BLAST search of hNDUFB3 against nr.

### Search parameters

| | |
|---|---|
| **Enzyme:** | Trypsin: cuts C-term side of KR unless next residue is P. |
| **Variable modifications:** | Acetyl (Protein N-term), Methyl (H), Oxidation (M), Propionamide (C) |
| **Mass values searched:** | 100 |
| **Mass values matched:** | 9 |

### Protein sequence coverage: 52%

Matched peptides shown in ***bold red***.

```
  1 AHEHGHEHGH HKMELPDYRQ WKIEGTPLET IQKKLAAKGL RDPWGRNEAW
 51 RYMGGFAKSV SFSDVFFKGF KWGFAAFVVA VGAEYYLESL NKDKKHHWRP
101 LEDYKDDDDK L
```

Unformatted sequence string: 111 residues (for pasting into other applications).

Sort by ◉ residue number ○ increasing mass ○ decreasing mass
Show ◉ matched peptides only ○ predicted peptides also

| Start – End | Observed | Mr(expt) | Mr(calc) | ppm | M | Peptide |
|---|---|---|---|---|---|---|
| 1 – 12 | 1454.6334 | 1453.6261 | 1453.6348 | -5.98 | 0 | -.AHEHGHEHGHHK.M + Acetyl (Protein N-term) |
| 1 – 12 | 1468.6513 | 1467.6440 | 1467.6504 | -4.38 | 0 | -.AHEHGHEHGHHK.M + Acetyl (Protein N-term); Methyl (H) |
| 1 – 19 | 2375.0284 | 2374.0211 | 2374.0410 | -8.35 | 1 | -.AHEHGHEHGHHKMELPDYR.Q + Acetyl (Protein N-term); Oxidation (M) |
| 13 – 19 | 923.4372 | 922.4299 | 922.4218 | 8.75 | 0 | K.MELPDYR.Q |
| 39 – 46 | 956.5028 | 955.4955 | 955.4988 | -3.43 | 1 | K.GLRDPWGR.N |
| 42 – 51 | 1286.5957 | 1285.5884 | 1285.5952 | -5.31 | 1 | R.DPWGRNEAWR.Y |
| 59 – 68 | 1162.5727 | 1161.5654 | 1161.5706 | -4.53 | 0 | K.SVSFSDVFFK.G |
| 96 – 105 | 1380.6719 | 1379.6646 | 1379.6734 | -6.41 | 0 | K.HHWRPLEDYK.D |
| 106 – 111 | 720.3141 | 719.3068 | 719.2974 | 13.1 | 1 | K.DDDDKL.- |

**No match to:** 700.3542, 711.1760, 713.1839, 715.1953, 722.3287, 738.2990, 744.3154, 755.1685, 757.1792, 760.2733, 791.4042, 799.2208, 804.2722, 834.4437, 845.4465, 850.4936, 857.3997, 873.3681, 876.2195, 879.2401, 881.4913, 884.2510, 900.2288, 910.3589, 927.3360, 933.3471, 944.5254, 949.3129, 965.2882, 993.9446, 1020.4996, 1028.5761, 1041.5427, 1041.9854, 1043.5857, 1058.5467, 1091.6247, 1095.6390, 1111.5589, 1127.5840, 1133.5277, 1137.5789, 1138.3507, 1146.5943, 1154.3243, 1176.5231, 1184.5953, 1192.5097, 1218.6775, 1303.5423, 1327.3820, 1334.5828, 1358.6747, 1393.6586, 1424.6779, 1433.6970, 1437.6175, 1449.7209, 1481.7487, 1546.8277, 1577.2206, 1580.7636, 1612.8111, 1640.8794, 1682.8523, 1696.7753, 1700.7914, 1727.7241, 1760.8432, 1768.9367, 1790.8748, 1852.8704, 1867.9676, 1872.8537, 1981.8572, 2045.9804, 2081.9561, 2109.0469, 2163.0275, 2201.0182, 2266.0118, 2273.1214, 2397.2529, 2427.1371, 2550.1777, 2848.2009, 2926.2441, 3058.3643, 3136.3217, 3153.3340, 3211.3171

Error: try setting browser cache to automatic.

Mascot: http://www.matrixscience.com/

# hNDUFB3 - *METTL9* KO cells

# MATRIX SCIENCE MASCOT Search Results

## Protein View: hNDUFB3

### hNDUFB3

| | |
|---|---|
| **Database:** | Shimazu |
| **Score:** | 27 |
| **Expect:** | 0.0036 |
| **Monoisotopic mass ($M_r$):** | 13052 |
| **Calculated pI:** | 7.30 |

Sequence similarity is available as **an NCBI BLAST search of hNDUFB3 against nr**.

### Search parameters

| | |
|---|---|
| **Enzyme:** | Trypsin: cuts C-term side of KR unless next residue is P. |
| **Variable modifications:** | Acetyl (Protein N-term), Methyl (H), Oxidation (M), Propionamide (C) |
| **Mass values searched:** | 103 |
| **Mass values matched:** | 6 |

### Protein sequence coverage: 46%

Matched peptides shown in ***bold red***.

```
  1 AHEHGHEHGH HKMELPDYRQ WKIEGTPLET IQKKLAAKGL RDPWGRNEAW
 51 RYMGGFAKSV SFSDVFFKGF KWGFAAFVVA VGAEYYLESL NKDKKHHWRP
101 LEDYKDDDDK L
```

Unformatted sequence string: **111 residues** (for pasting into other applications).

Sort by ◉ residue number ○ increasing mass ○ decreasing mass
Show ◉ matched peptides only ○ predicted peptides also

| Start – End | Observed | Mr(expt) | Mr(calc) | ppm | M | Peptide |
|---|---|---|---|---|---|---|
| 1 – 12 | 1454.6522 | 1453.6449 | 1453.6348 | 6.98 | 0 | -.AHEHGHEHGHHK.M<br>+ Acetyl (Protein N-term) |
| 1 – 19 | 2375.0260 | 2374.0187 | 2374.0410 | -9.38 | 1 | -.AHEHGHEHGHHKMELPDYR.Q<br>+ Acetyl (Protein N-term); Oxidation (M) |
| 39 – 46 | 956.5167 | 955.5094 | 955.4988 | 11.1 | 1 | K.GLRDPWGR.N |
| 42 – 51 | 1286.6262 | 1285.6190 | 1285.5952 | 18.5 | 1 | R.DPWGRNEAWR.Y |
| 59 – 68 | 1162.6241 | 1161.6168 | 1161.5706 | 39.7 | 0 | K.SVSFSDVFFK.G |
| 96 – 105 | 1380.6889 | 1379.6816 | 1379.6734 | 5.94 | 0 | K.HHWRPLEDYK.D |

**No match to:** 700.2144, 705.2009, 707.2111, 709.1979, 711.1893, 713.2046, 715.2101, 717.2103, 721.2010, 722.3422, 725.2131, 729.2088, 731.1886, 733.1861, 735.1998, 738.3120, 739.1796, 742.2227, 746.2230, 749.1777, 751.1958, 752.2130, 755.1889, 757.1949, 761.2027, 772.2131, 775.1946, 777.1738, 779.1919, 787.2339, 792.2448, 793.1594, 795.1794, 797.2278, 799.2345, 804.2835, 810.2465, 812.2421, 814.2429, 817.2516, 826.2734, 832.2589, 836.2495, 838.2517, 840.2592, 845.4700, 853.2517, 856.2490, 857.4268, 858.2623, 860.2485, 862.2415, 876.2382, 878.2457, 880.2427, 882.2407, 884.2510, 894.2334, 898.2481, 900.2405, 902.2498, 904.2567, 909.3622, 911.3585, 917.2564, 920.2343, 922.2360, 925.2894, 927.3548, 933.3551, 939.2384, 941.2578, 943.2575, 946.2538, 949.3226, 966.2500, 986.2839, 989.5860, 1001.2954, 1020.5191, 1043.5978, 1089.2902, 1091.2925, 1135.2946, 1138.3624, 1154.3430, 1180.6408, 1358.6929, 1449.7350, 1546.8385, 1580.7775, 1682.8765, 2081.9671, 2273.1342, 2926.2746, 3153.3587, 3211.3504

Error: try setting browser cache to automatic.

# mS100A9 - WT neutrophils

# MATRIX SCIENCE MASCOT Search Results

## Protein View: S100A9

### S100A9

| | |
|---|---|
| **Database:** | Shimazu |
| **Score:** | 29 |
| **Expect:** | 0.0025 |
| **Monoisotopic mass ($M_r$):** | 12909 |
| **Calculated pI:** | 6.73 |

Sequence similarity is available as an NCBI BLAST search of S100A9 against nr.

### Search parameters

| | |
|---|---|
| **Enzyme:** | Lys-C/P: cuts C-term side of K. |
| **Variable modifications:** | Acetyl (Protein N-term), Methyl (H), Oxidation (M), Propionamide (C) |
| **Mass values searched:** | 73 |
| **Mass values matched:** | 8 |

### Protein sequence coverage: 75%

Matched peptides shown in ***bold red***.

```
  1 ANKAPSQMER SITTIIDTFH QYSRKEGHPD TLSKKEFRQM VEAQLATFMK
 51 KEKRNEALIN DIMEDLDTNQ DNQLSFEECM MLMAKLIFAC HEKLHENNPR
101 GHGHSHGKGC GK
```

Unformatted sequence string: 112 residues (for pasting into other applications).

Sort by ◉ residue number ○ increasing mass ○ decreasing mass
Show ◉ matched peptides only ○ predicted peptides also

| Start – End | Observed | Mr(expt) | Mr(calc) | ppm | M | Peptide |
|---|---|---|---|---|---|---|
| 4 – 25 | 2609.3703 | 2608.3630 | 2608.3067 | 21.6 | 0 | K.APSQMERSITTIIDTFHQYSRK.E |
| 4 – 25 | 2625.3602 | 2624.3529 | 2624.3017 | 19.5 | 0 | K.APSQMERSITTIIDTFHQYSRK.E + Oxidation (M) |
| 36 – 50 | 1828.9432 | 1827.9359 | 1827.9011 | 19.0 | 0 | K.EFRQMVEAQLATFMK.K |
| 36 – 50 | 1844.9358 | 1843.9285 | 1843.8961 | 17.6 | 0 | K.EFRQMVEAQLATFMK.K + Oxidation (M) |
| 36 – 50 | 1860.9234 | 1859.9161 | 1859.8910 | 13.5 | 0 | K.EFRQMVEAQLATFMK.K + 2 Oxidation (M) |
| 54 – 85 | 3774.7528 | 3773.7455 | 3773.6714 | 19.6 | 0 | K.RNEALINDIMEDLDTNQDNQLSFEECMMLMAK.L |
| 94 – 108 | 1690.8643 | 1689.8570 | 1689.8196 | 22.1 | 0 | K.LHENNPRGHGHSHGK.G + Methyl (H) |
| 94 – 108 | 1704.8793 | 1703.8721 | 1703.8353 | 21.6 | 0 | K.LHENNPRGHGHSHGK.G + 2 Methyl (H) |

**No match to:** 706.2132, 711.1810, 713.2066, 715.2074, 722.3378, 730.2127, 733.1975, 738.3161, 746.2200, 757.1942, 778.2077, 799.2448, 804.2929, 805.3040, 818.3945, 845.9361, 846.4402, 905.9933, 914.9774, 915.4780, 923.4734, 923.9704, 927.3566, 949.3355, 965.3110, 993.9742, 1186.2501, 1211.6538, 1305.1877, 1305.6892, 1310.7139, 1313.6894, 1693.9257, 1734.0226, 1756.9372, 1789.9392, 1809.9809, 1819.9132, 1831.9515, 1850.9279, 1866.8951, 1905.9923, 1924.0826, 1931.1767, 1966.0817, 2105.1484, 2136.2733, 2150.2877, 2217.3516, 2238.1289, 2370.5115, 2429.4220, 2540.2811, 2552.5062, 2564.3858, 2569.8678, 2593.4361, 2608.8524, 2631.3362, 2647.3227, 2821.6293, 2863.5358, 3979.0959, 4107.2052, 4504.2375

Error: try setting browser cache to automatic.

Mascot: http://www.matrixscience.com/

# mS100A9 - *Mettl9* KO neutrophils

# MATRIX SCIENCE MASCOT Search Results

## Protein View: S100A9

### S100A9

| | |
|---|---|
| **Database:** | Shimazu |
| **Score:** | 34 |
| **Expect:** | 0.00087 |
| **Monoisotopic mass ($M_r$):** | 12909 |
| **Calculated pI:** | 6.73 |

Sequence similarity is available as **an NCBI BLAST search of S100A9 against nr**.

### Search parameters

| | |
|---|---|
| **Enzyme:** | Lys-C/P: cuts C-term side of K. |
| **Variable modifications:** | Acetyl (Protein N-term), Methyl (H), Oxidation (M), Propionamide (C) |
| **Mass values searched:** | 100 |
| **Mass values matched:** | 7 |

### Protein sequence coverage: 75%

Matched peptides shown in ***bold red***.

```
  1 ANKAPSQMER SITTIIDTFH QYSRKEGHPD TLSKKEFRQM VEAQLATFMK
 51 KEKRNEALIN DIMEDLDTNQ DNQLSFEECM MLMAKLIFAC HEKLHENNPR
101 GHGHSHGKGC GK
```

Unformatted sequence string: **112 residues** (for pasting into other applications).

Sort by ◉ residue number ○ increasing mass ○ decreasing mass
Show ◉ matched peptides only ○ predicted peptides also

| Start – End | Observed | Mr(expt) | Mr(calc) | ppm | M | Peptide |
|---|---|---|---|---|---|---|
| 4 – 25 | 2609.3598 | 2608.3526 | 2608.3067 | 17.6 | 0 | K.APSQMERSITTIIDTFHQYSRK.E |
| 4 – 25 | 2625.3443 | 2624.3370 | 2624.3017 | 13.5 | 0 | K.APSQMERSITTIIDTFHQYSRK.E + Oxidation (M) |
| 36 – 50 | 1828.9421 | 1827.9348 | 1827.9011 | 18.4 | 0 | K.EFRQMVEAQLATFMK.K |
| 36 – 50 | 1844.9292 | 1843.9220 | 1843.8961 | 14.1 | 0 | K.EFRQMVEAQLATFMK.K + Oxidation (M) |
| 36 – 50 | 1860.9105 | 1859.9033 | 1859.8910 | 6.61 | 0 | K.EFRQMVEAQLATFMK.K + 2 Oxidation (M) |
| 54 – 85 | 3774.7104 | 3773.7031 | 3773.6714 | 8.40 | 0 | K.RNEALINDIMEDLDTNQDNQLSFEECMMLMAK.L |
| 94 – 108 | 1676.8459 | 1675.8386 | 1675.8040 | 20.7 | 0 | K.LHENNPRGHGHSHGK.G |

**No match to:** 706.2167, 707.2064, 711.1945, 713.2087, 715.2168, 722.3437, 730.2181, 732.2099, 734.2208, 738.3184, 757.2108, 772.2157, 776.2389, 778.2245, 799.2442, 804.2990, 805.3010, 806.2990, 826.2671, 836.2586, 842.2545, 857.4176, 905.4946, 905.9678, 914.9813, 915.4843, 922.9696, 927.3566, 949.3360, 965.3135, 993.9724, 1087.6511, 1106.6925, 1154.3542, 1211.6566, 1272.1280, 1294.6706, 1305.1919, 1306.6942, 1313.1788, 1411.3178, 1411.8183, 1432.2672, 1432.7625, 1449.9515, 1511.7569, 1529.7653, 1734.0181, 1756.9514, 1789.9481, 1809.9779, 1813.9223, 1819.9035, 1831.9396, 1840.8997, 1847.9015, 1850.9094, 1866.8895, 1905.9971, 1966.0770, 2105.1411, 2157.1814, 2217.3452, 2238.1279, 2253.1418, 2254.6640, 2260.6543, 2261.1212, 2370.4973, 2500.6276, 2525.2714, 2540.2726, 2544.3703, 2556.2510, 2562.2405, 2569.5743, 2578.2068, 2593.4113, 2647.2886, 2821.6248, 2837.6075, 2844.5424, 2863.0178, 2863.5203, 3236.6621, 4107.1709, 4488.1626, 4502.7077, 4504.2202, 4520.2128, 4527.1975, 4542.1692, 4873.2814

Error: try setting browser cache to automatic.

**Mascot: http://www.matrixscience.com/**
