# Supplementary material for: The methyltransferase METTL9 mediates pervasive 1-methylhistidine modification in mammalian proteomes
Source: Nat Commun. 2021 Feb 9;12:891. doi: 10.1038/s41467-020-20670-7 (PMC7873184; doi:10.1038/s41467-020-20670-7)
Supplement: Supplementary file 6 — Reporting Summary [file 41467_2020_20670_MOESM6_ESM.pdf]

nature research

Corresponding author(s): Pål Ø. Falnes, Yoichi Shinkai, Albert Jeltsch

Last updated by author(s): Dec 1, 2020

Reporting Summary

Nature Research wishes to improve the reproducibility of the work that we publish. This form provides structure for consistency and transparency in reporting. For further information on Nature Research policies, see our Editorial Policies and the Editorial Policy Checklist.

Statistics

For all statistical analyses, confirm that the following items are present in the figure legend, table legend, main text, or Methods section.

n/a	Confirmed	
☐	☒	The exact sample size (*n*) for each experimental group/condition, given as a discrete number and unit of measurement
☐	☒	A statement on whether measurements were taken from distinct samples or whether the same sample was measured repeatedly
☐	☒	The statistical test(s) used AND whether they are one- or two-sided<br>*Only common tests should be described solely by name; describe more complex techniques in the Methods section.*
☒	☐	A description of all covariates tested
☒	☐	A description of any assumptions or corrections, such as tests of normality and adjustment for multiple comparisons
☐	☒	A full description of the statistical parameters including central tendency (e.g. means) or other basic estimates (e.g. regression coefficient) AND variation (e.g. standard deviation) or associated estimates of uncertainty (e.g. confidence intervals)
☐	☒	For null hypothesis testing, the test statistic (e.g. *F*, *t*, *r*) with confidence intervals, effect sizes, degrees of freedom and *P* value noted<br>*Give P values as exact values whenever suitable.*
☒	☐	For Bayesian analysis, information on the choice of priors and Markov chain Monte Carlo settings
☒	☐	For hierarchical and complex designs, identification of the appropriate level for tests and full reporting of outcomes
☒	☐	Estimates of effect sizes (e.g. Cohen's *d*, Pearson's *r*), indicating how they were calculated

Our web collection on statistics for biologists contains articles on many of the points above.

Software and code

Policy information about availability of computer code

Data collection	No custom software was used for data collection. Xcalibur (ver. 4.0/4.1) was used for acquiring mass spectrometry raw data.
Data analysis	No custom software was used. The following standard software was used.<br>Analysis of protein mass spectrometry data: Proteome Discoverer™ (ver. 1.4) ,SEQUEST™, MaxQuant (ver 1.6.3.3), Mascot (ver 2.6.0/2.7.0), Xcalibur Qual Browser (ver. 4.1)<br>Structure prediction: RaptorX. Multiple sequence alignment: Jalview, ver. 2.11.0.

For manuscripts utilizing custom algorithms or software that are central to the research but not yet described in published literature, software must be made available to editors and reviewers. We strongly encourage code deposition in a community repository (e.g. GitHub). See the Nature Research guidelines for submitting code & software for further information.

Data

Policy information about availability of data

All manuscripts must include a data availability statement. This statement should provide the following information, where applicable:

- Accession codes, unique identifiers, or web links for publicly available datasets
- A list of figures that have associated raw data
- A description of any restrictions on data availability

The immunoprecipitation mass spectrometry proteomics data have been deposited to the ProteomeXchange Consortium via the PRIDE52 partner repository with the dataset identifier PXD016408; http://proteomecentral.proteomexchange.org/cgi/GetDataset?ID=PXD016408
The ProSeAm SILAC screen data have been deposited at ProteomeXchange, ID: PXD016823; http://proteomecentral.proteomexchange.org/cgi/GetDataset?ID=PXD016823
The DNAJB12 methylation data have been deposited to ProteomeXchange, ID: PXD020010; http://proteomecentral.proteomexchange.org/cgi/GetDataset?

ID=PXD020010
NDUFB3 and S100A9 methylation data are available via ProteomeXchange with identifier PXD022067; https://www.ebi.ac.uk/pride/archive/projects/PXD022067
The following publicly available databases were used: UniProt, (https://www.uniprot.org/) for obtaining protein sequences, and RCSB Protein Data Bank (RCSB PDB) (https://www.rcsb.org/) for obtaining protein structures.

The source data underlying Figs 1a, c, f, 2c-d, 3d, 4a, d and 5b-c, and Supplementary Figs 9a-b and 10a-b are provided as a Source Data file.

Field-specific reporting

Please select the one below that is the best fit for your research. If you are not sure, read the appropriate sections before making your selection.

☒ Life sciences ☐ Behavioural & social sciences ☐ Ecological, evolutionary & environmental sciences

For a reference copy of the document with all sections, see nature.com/documents/nr-reporting-summary-flat.pdf

Life sciences study design

All studies must disclose on these points even when the disclosure is negative.

Sample size	No sample-size calculations were performed, as this was not considered necessary/relevant. Sample sizes (i.e. numbers of parallells and replicates) were chosen sufficiently large for the observed differences (between sample sets) to be significant and robust, but at the same time not larger than what was required for efficient and accurate exectution of the experiments (e.g. from a logistics perspective).
Data exclusions	No data were excluded from the analyses.
Replication	All key findings were replicated in independent experiments, typically 2-3 times. The number of experiments performed is indicated in the figure legends.
Randomization	This is not very relevant for this study. But it should be mentioned that parallels of the same sample were distributed throughout the experimental series, thereby potentially avoiding (handling) time or position (e.g. on a multiwell plate) effects.
Blinding	Blinding was not considered necessary, as the data represent biochemical measurements, and do not result from potentially subjective observations.

Reporting for specific materials, systems and methods

We require information from authors about some types of materials, experimental systems and methods used in many studies. Here, indicate whether each material, system or method listed is relevant to your study. If you are not sure if a list item applies to your research, read the appropriate section before selecting a response.

Materials & experimental systems

n/a	Involved in the study	
☐	☒	Antibodies
☐	☒	Eukaryotic cell lines
☒	☐	Palaeontology and archaeology
☐	☒	Animals and other organisms
☒	☐	Human research participants
☒	☐	Clinical data
☒	☐	Dual use research of concern

Methods

n/a	Involved in the study	
☒	☐	ChIP-seq
☒	☐	Flow cytometry
☒	☐	MRI-based neuroimaging

Antibodies

Antibodies used	ANTI-FLAG M2 Monoclonal Antibody (SIGMA, F1804); anti-ZIP7 antibody (Protein Tech, 19429-1-AP); anti-DNAJB12 antibody (Proteintech, 16780-1-AP)
Validation	https://www.sigmaaldrich.com/catalog/product/sigma/f1804?lang=en&region=US<br>https://www.thermofisher.com/antibody/product/ZIP7-Antibody-Polyclonal/19429-1-AP<br>https://www.thermofisher.com/antibody/product/DNAJB12-Antibody-Polyclonal/16780-1-AP

Eukaryotic cell lines

Policy information about cell lines

Cell line source(s)	METTL9 KO HAP-1 cells (cat. no. HZGHC004343c010) and corresponding "wild-type" control cells were from Horizon Discovery. Sf9 cells and Flp-In T-REx HEK-293 cells were from Invitrogen (now Thermo-Fischer). HeLa and HEK293T cells were from ATCC.
Authentication	HAP-1 METTL9 KO cells were authenticated by DNA sequencing. Flp-In T-REx HEK-293 were (indirectly) authenticated through their ability to accept a transgene through site-specific recombination. HEK-293, Sf9, and HeLa cells were not authenticated, but, given the type of experiments the cells were used for, such identification was not considered critical.
Mycoplasma contamination	Cells were regulary tested for mycoplasma infection, or, alternatively, a fresh aliqout of cells was used for each experiment (Sf9 cells).
Commonly misidentified lines (See ICLAC register)	No commonly misidentified cell lines were used.

Animals and other organisms

Policy information about studies involving animals; ARRIVE guidelines recommended for reporting animal research

Laboratory animals	C57BL/6J mice were obtained from CLEA Japan, Inc., and used to generate METTL9 knock-out mice. Studies were performed with 7 month old female mice.
Wild animals	The study did not involve wild animals
Field-collected samples	The study did not involve samples collected from the field
Ethics oversight	All experiments involving mice were carried out according to protocols approved by the Animal Experiment Committee of the RIKEN Center for Brain Science.

Note that full information on the approval of the study protocol must also be provided in the manuscript.
